# Tunable structural colors based on grayscale lithography and conformal coating of VO_2_

**DOI:** 10.1515/nanoph-2024-0546

**Published:** 2025-01-17

**Authors:** Xiaochen Zhang, Haozhe Sun, Yuan Li, Jianhua Hao, Qinghua Liang, Yongyue Zhang, Yang Wang, Xiaowei Li, Xinping Zhang, He Ma, Jiafang Li

**Affiliations:** Key Lab of Advanced Optoelectronic Quantum Architecture and Measurement (Ministry of Education), Beijing Key Lab of Nanophotonics & Ultrafine Optoelectronic Systems, and School of Physics, 47833 Beijing Institute of Technology, Beijing 100081, China; Institute of Information Photonics Technology, School of Physics and Optoelectronic Engineering, Beijing University of Technology, Beijing 100124, China; Laser Micro/Nano Fabrication Laboratory, School of Mechanical Engineering, 47833 Beijing Institute of Technology, Beijing 100081, China

**Keywords:** structural colors, vanadium dioxide, phase change materials, grayscale exposure, electrical control

## Abstract

Structural colors generated by optical micro-/nanostructures offer a notable advantage over traditional chemical pigments, including higher purity, greater brightness, resistance to fading, and enhanced environmental friendliness. However, achieving dynamically switchable color displays with high performances and without resorting to complex nanofabrication methods remain a challenge. Here, we present a simple method using grayscale lithography and conformal coating to create Salisbury screen (SS) cavities with variable resonant wavelengths, enabling the formation of tunable colorful patterns. The dynamic color display is achieved through the phase change of vanadium dioxide (VO_2_) nanostructures under electrothermal effects. At a low actuation voltage of 1.4 V, high performances of color switching such as high sensitivity, fast speed, high repeatability, and wide-view angle are achieved. The tunable structural colors, featuring a simple preparation process and high-speed switching, represent a promising alternative for applications such as thermal sensors, security information encryption, and dynamic full-color displays.

## 1 Introduction

Structural colors are a fascinating and vibrant phenomenon that fundamentally differs from chemical colors [1], [2]. While chemical colors are produced by the absorption of specific wavelengths of light by materials, structural colors arise from the physical interaction of light with microscopic and nanoscopic structures on the surface of the material. In recent years, significant advances have been achieved in metasurface-based structural colors [3], [4], [5]. These metasurface interactions include thin-film interference effects [6], [7], [8], [9], diffraction from periodic structures [4], [5], and wavelength-selective scattering produced by particles, all of which contribute to the generation of brighter and more vivid colors [10]. For instance, the color gamut area of silicon metasurface structural colors reaches a relatively high proportion, increasing the spatial resolution to the diffraction limit [4]. These colors, formed through the manipulation of nanoscale structures, offer enhanced stability and resistance to fading, making them ideal for advanced display technologies. However, many methods for creating structural colors involve complex fabrication processes, limiting their practical application and integration on a device scale. One notable approach for producing structural colors is the metal–insulator–metal Fabry–Pérot (MIMFP) cavity, a fundamental and widely used design [11], [12]. An asymmetric variant of this cavity is the Salisbury screen (SS) structure, which features a reflective metal layer at the bottom, a dielectric layer in the middle, and a semi-transparent thin metal layer on top [13]. While static structural colors have achieved high-resolution, high-saturation, and wide-gamut color display applications [14], [15], [16], the potential advancements for dynamic color displays are highly appealing. The challenge lies in developing adjustable structural colors that can meet the high frame rate requirements necessary for dynamic color displays. Due to constraints in modulation mechanisms and methods, existing adjustable structural colors fall short in this regard. For instance, stimuli-responsive liquid crystals (LCs) can provide dynamic and reversible tunability with rapid color switching upon external stimuli [8]. However, the slow response time of LCs and their birefringence characteristics restrict their application in high-speed displays.

In the current studies, external stimuli for changing structural colors include electrical signals [8], [17], mechanical deformation [18], [19], temperature changes [6], [20], [21], and chemical interactions [22], [23]. Electrical tuning is a dynamic modulation method that is relatively easy to integrate and apply in practice. However, adjustable structural colors with fast response, low power dissipation, and long lifetime are still challenging. On the other hand, electrically driven phase change materials have sparked numerous innovations and breakthroughs in the field of tunable nanophotonics [8], [24], [25], [26]. Vanadium dioxide (VO_2_) is a phase change material that undergoes the reversible phase transition from an insulating to a metallic state near 68 °C, which significantly alters its optical properties [27], [28]. This transition can be triggered rapidly by an external stimuli such as heat [29], [30], [31], electric [25], [32], [33], [34], [35], [36], [37], and light [38], [39], allowing for high-speed and reversible changes in colors. These features make VO_2_ a promising material for advanced display technologies [20], [32], [40], smart windows [41], [42], [43], [44], switchable holography [30], and other applications requiring controllable and responsive color changes. Actively tunable structural colors hold great potential for applications in adaptive displays [45], smart textiles [46], [47], and sensors [21], [48], [49]. However, the huge thermal capacity often limits the high-speed modulation and power dissipation reduction of the device, and the ultrathin pixel structure faces significant challenges compared to passive structured color devices.

In this work, we demonstrate a strategy of tunable structural colors based on conformal VO_2_-coated Salisbury screen cavities with different resonant wavelengths. The hybrid structures are fabricated by employing an electron beam lithography (EBL)-based grayscale exposure and a conformal coating method to combine vanadium dioxide (VO_2_) and polymethyl methacrylate (PMMA) on a gold-coated ultra-thin silicon nitride (Si_3_N_4_) windows. Versatile structural colors formed by the freestanding hybrid nanostructures exhibit high performance of dynamic tunability when actuated by a low voltage of 1.4 V. Prototype applications of electrothermal color display and thermal-induced “converging” colors are demonstrated. The combination of a straightforward fabrication process, fast modulation speed, and low triggering power underscores the broad application prospects of this technology in dynamic color displays, which may be useful to achieve smooth display effects with high frame rates and provide an attractive solution for advanced display technologies.

## 2 Results

### 2.1 Structural design and nanofabrication

**Figure 1:**
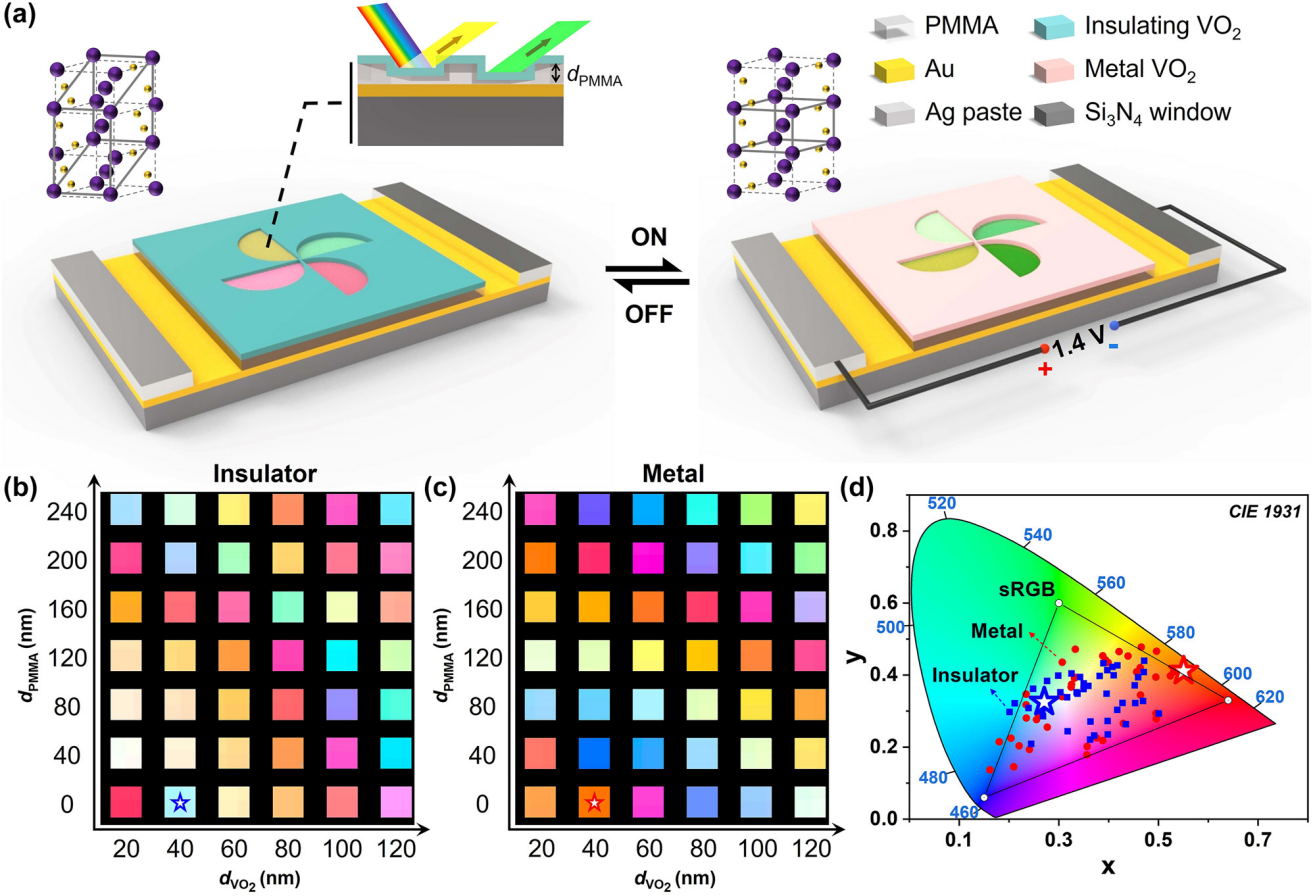
Schematic of Salisbury screen (SS) cavities for color display. (a) Schematic diagram of the VO_2_/PMMA/Au structures, illustrating the structural design and operational principles based on SS cavities. The inset displays the transformation of lattice structures during the phase transition of VO_2_, with large vanadium atoms in purple and small oxygen atoms in yellow. (b) and (c) Simulated color plates of SS cavity with VO_2_ and PMMA layers of different thickness as noted, in the insulator phase and metal phase of VO_2_, respectively. Each color square is calculated based on the simulated reflection spectra of the structure. (d) Plot of the simulated colors of (b) and (c) in CIE 1931 space. The positions of the stars correspond to specific structures depicted in (b) and (c), indicating the structural parameters with the largest color changes.

The schematic diagram of the structural color display and switching under external voltage stimulation is shown in Figure 1(a). The device consists of a VO_2_ layer on top, a PMMA layer in the middle, and a thick Au film on the bottom. Due to the ultralow thermal capacity of the freestanding multilayer film, the trigger voltage of the structure is only 1.4 V. During the transition from insulator to metal, the lattice structure of VO_2_ changes from monoclinic phase (M1-VO_2_) to rutile phase (R-VO_2_), as shown in the inset of Figure 1(a). To investigate the color-changing ability of VO_2_, the reflection spectra in both insulating and metallic states are simulated (see Supplementary Figure S1(b) and (c)). This simulation provides insights into the changes in the optical properties of VO_2_ during its phase transition, which is crucial for designing devices that leverage its dynamic tunable color capabilities. For example, by analyzing the reflection spectra, one can predict the colors produced during the phase change and optimize the VO_2_ layer for various applications in dynamic color display, which also enables the fine-tuning of the optical responses of VO_2_ to enhance the overall functionality and versatility of dynamic display technologies. Based on the simulated reflection spectra, Figure 1(b) and (c) plot the versatile colors and their transitions by varying the structural parameters. For the structure with 40-nm-thick VO_2_ and without the PMMA layer (marked by the stars), Figure 1(b) and (c) show that the change in refractive index during phase transition promotes the significant color change from blue to orange. Furthermore, color mixing is a common problem in structural colors, mainly due to nonresonant components and broad resonance peaks. Therefore, color purity is an important characterization parameter that represents the purity of color without mixing white light or other colors. This purity can be calculated by using the following equation [50]:

(1) $$color\;purity=\frac{\sqrt{{\left(x-x_{i}\right)}^{2}+{\left(y-y_{i}\right)}^{2}}}{\sqrt{{\left(x_{d}-x_{i}\right)}^{2}+{\left(y_{d}-y_{i}\right)}^{2}}}\times 100\;\%,$$

where (*x*, *y*), (*x*_*i*_, *y*_*i*_), and (*x*_*d*_, *y*_*d*_) represent the corresponding CIE coordinates of the samples, standard white light (0.310, 0.316), and dominant wavelength, respectively. The obtained orange purity of the nanostructure is 92 %. The color gamut of standard red-green-blue (sRGB) color space is marked by the black triangle. By adjusting the thickness of the VO_2_ and PMMA layers, the insulating and metallic VO_2_ phases can achieve a comprehensive color gamut, covering 41 % and 67 % of the sRGB color space, respectively, as shown in Figure 1(d). This tunability highlights the substantial potential of VO_2_ for use in applications requiring dynamic and vivid color displays.

**Figure 2:**
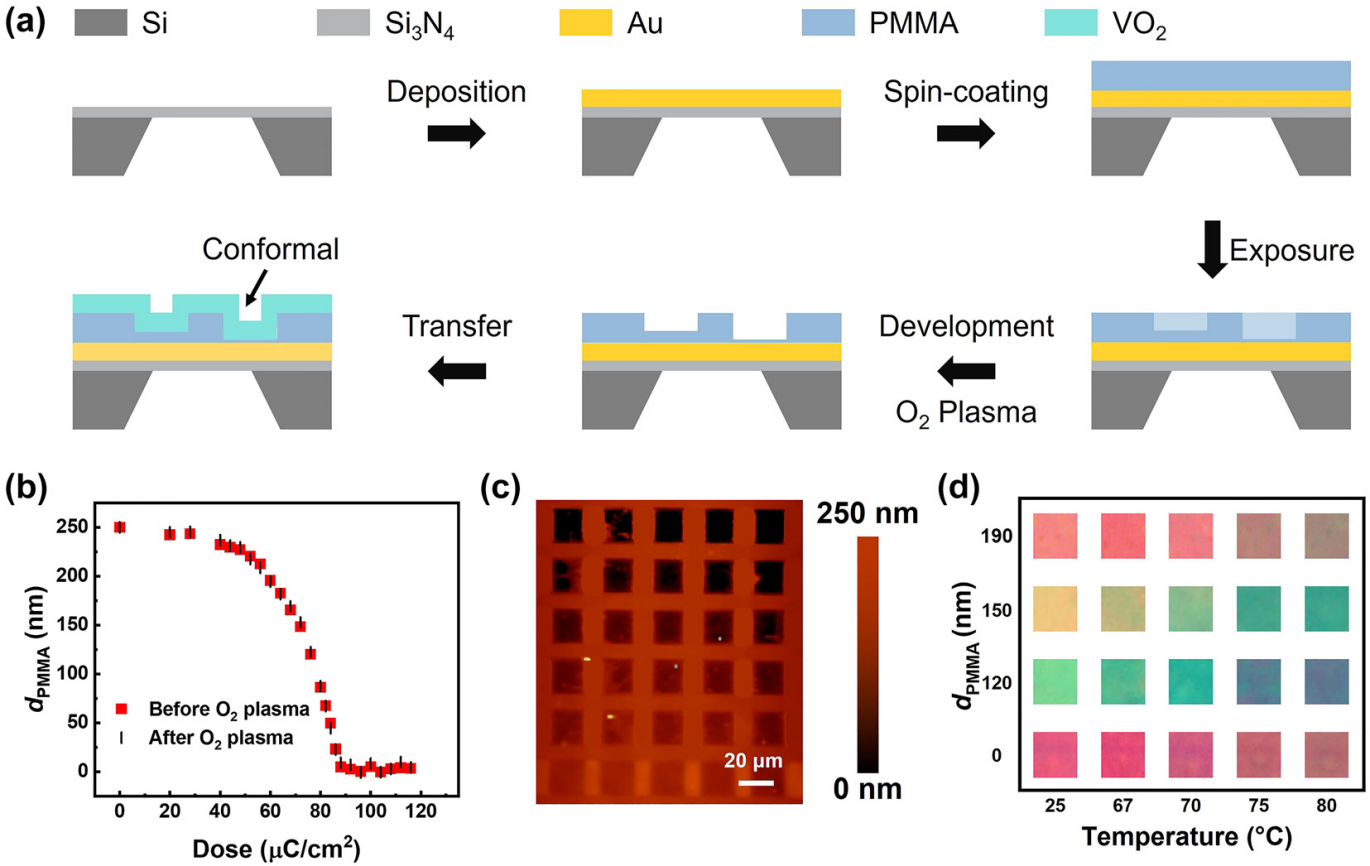
Fabrication method of gray scale exposure. (a) Flowchart of the grayscale exposure with EBL and conformal coating with VO_2_. (b) Thickness of PMMA as a function of the exposure dose. (c) Topographic image measured by AFM, showing square structures with different height, which clearly shows the conformal coating. (d) Captured images of the square structures with different height when temperature varies from 25 to 80 °C.

To realize such structural designs, the fabrication process includes three main steps: electron beam (E-beam) deposition of gold, EBL grayscale exposure of nanopatterns, and VO_2_ film transfer [27]. The high excitation threshold is mainly due to the large thermal capacity of the hard substrate material. In this study, a suspended thin-film configuration with ultralow thermal capacity and small thickness is employed to reduce the excitation threshold. As shown in the schematic diagram of Figure 2(a), a 100-nm-thick gold electrode is lithographically patterned on the Si_3_N_4_ window to suppress light transmission (the microscope image of the electrode pattern is shown in Figure S2(a). Then, the lossless PMMA films of different thickness are prepared under different exposure doses on the top of the Au layer. PMMA is chosen since it is a high-resolution electron beam resist with high sensitivity to electron beam dose and is thus desirable for applications with different thicknesses. Finally, the VO_2_ film was conformally transferred onto the PMMA layer to form the SS cavity with different spacer thicknesses (*d*_PMMA_). The freestanding VO_2_ film before transfer is shown in Figure S2(b) and (c). The transfer method is primarily employed because the performance of VO_2_ film is significantly influenced by the substrate. Additionally, the high temperatures necessary for the crystallization of the VO_2_ phase, typically exceeding 500 °C, can damage PMMA. This makes it unsuitable for direct deposition on PMMA polymer materials. To precisely control the height of the nanostructure, the relationship between the electron beam dose and the remaining PMMA thickness is measured by the stylus profiler. The resolved data in Figure 2(b) indicate that the thickness of the PMMA decreases gradually when the electron beam dose is more than 40 μC/cm^2^ and decreases significantly to 0 nm when the dose reaches ∼90 μC/cm^2^. To enhance the hydrophilicity of the PMMA surface prior to VO_2_ film transfer, we treated it with oxygen plasma. As demonstrated in Figure 2(b), after PMMA is treated with low-power oxygen plasma for 10 s, the PMMA thickness remains nearly unchanged (decreases by an average of 1.1 nm), resulting in negligible effect on the resonance wavelength of the FP cavity. Such a method allows us to fine-tune the structural parameters, ensuring precise control over the optical properties of the device while maintaining the integrity of the PMMA layer.

The local height of VO_2_ film after transferred onto the PMMA is further precisely characterized by atomic force microscopy (AFM), as the images shown in Figure 2(c). It can be seen that from the first row to the fifth row, the local contrast increases gradually, indicating a decreased PMMA thickness in the square and confirming the conformal coating of VO_2_ with PMMA after the transfer process, which is critical for the high-resolution color displays. As a typical stimulus-responsive phase change material, VO_2_ shows the obvious response to external electric, thermal, and light stimulation, making it one of the most promising materials for tunable structural colors. To test this capability, the hybrid structures are heated by a hot and cold stage and the changes in structural colors with temperature are plotted in Figure 2(d), showing dynamically tunable structural colors by adjusting the applied temperature. In addition, we observed that the VO_2_/PMMA/Au multilayer structure exhibits continuous color tunability from room temperature to 80 °C.

### 2.2 Electrothermally tunable structural colors

**Figure 3:**
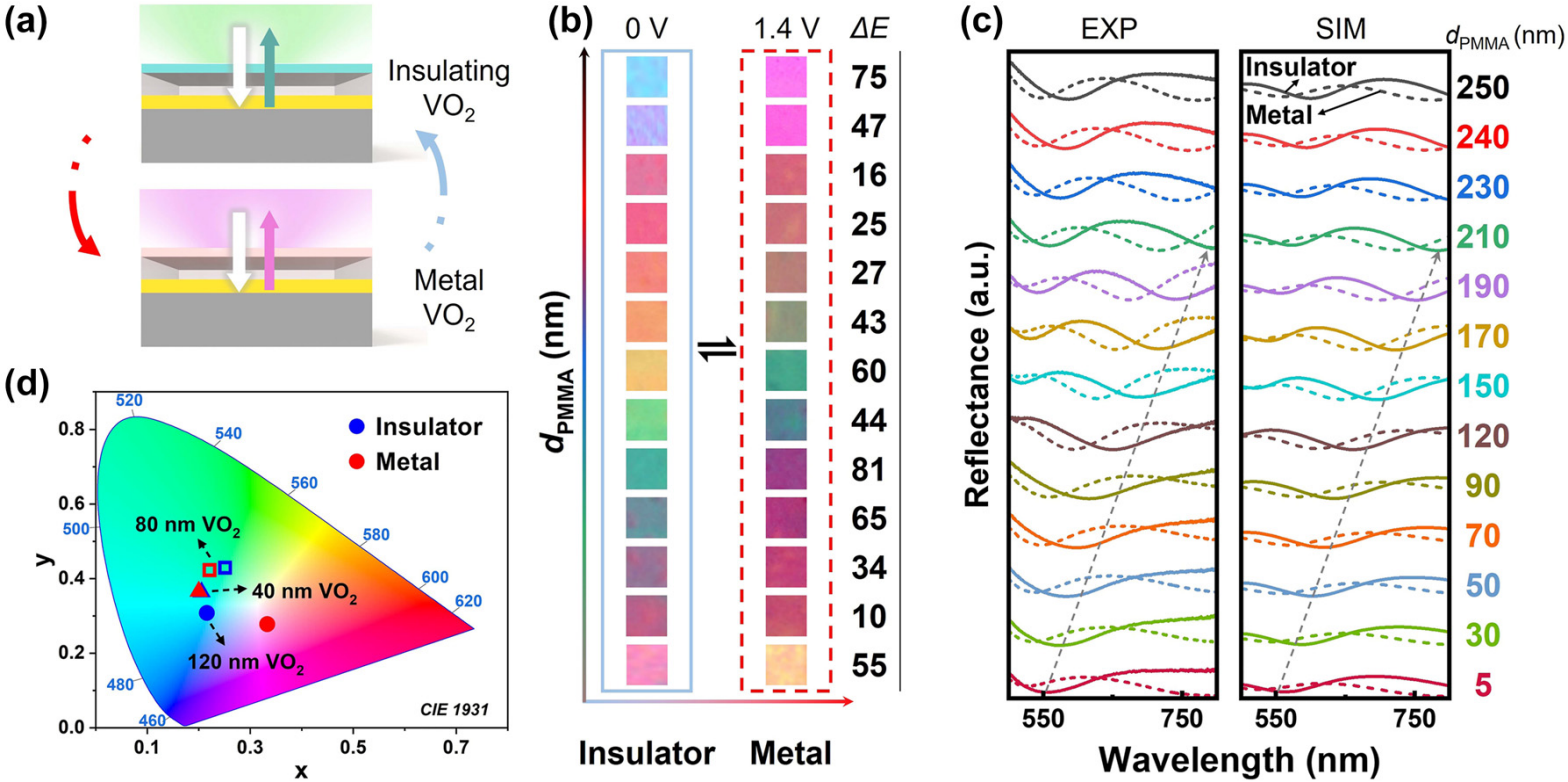
Dynamically tunable structural colors achieved through electric stimulus. (a) Schematic of the working principle of the SS cavity structure before and after the phase change of VO_2_. (b) Color images of the squares with PMMA of different the thickness as noted at the applied voltage of 0 and 1.4 V. The thickness of the VO_2_ thin film is 120 nm. The blue solid frame and red dotted line frame represent the insulating and metal phases of VO_2_, respectively. Δ*E* in the right column means the color difference. (c) Measured and simulated reflection spectra of SS cavity that consist of a 120-nm-thick VO_2_ film under different *d*_PMMA_. Both spectra exhibit a red shift as *d*_PMMA_ increases, as indicated by the gray dashed arrows. (d) Measured structural colors in CIE 1931 space when the thickness of VO_2_ is 40, 80, and 120 nm under *d*_PMMA_ = 250 nm.

The asymmetric SS MIMFP cavity is very desirable to enhance the structural color performance by leveraging the principles of interference. In this design, multiple reflections between parallel surfaces create standing wave patterns that selectively reflect specific wavelengths, thereby producing bright colors. The thickness of the thin metal layer on top of the SS cavity is less than the skin depth of the incident light, allowing part of the light to penetrate the cavity. Figure 3(a) presents the schematic design of our reflective device, where the color change in the SS structure is attributed to the VO_2_ phase transition. Based on the SS cavity, by controlling the EBL exposure process, we can adjust the thickness of the PMMA in the middle layer to adjust the length of the SS cavity and thus regulate the displayed colors. The coherent accumulation of the reflection in the FP cavity satisfies the resonant wavelength condition [51], which can be expressed as:

(2) $$\lambda =4\pi nd_{\text{PMMA}}\cos\theta /\left(2k\pi -\varphi_{1}-\varphi_{2}\right),$$

where *n* is the effective refractive index of dielectric layers consisting of VO_2_, *d*_PMMA_ is the thickness of PMMA layers, *θ* is the incident angle, and *φ*_1_ and *φ*_2_ represent the phase shift of the reflection coefficients at the two interfaces of the FP cavity.

In this model, when VO_2_ undergoes a phase transition by external stimuli, its refractive index will experience a significant change, making VO_2_ highly promising for applications in tunable color displays. To validate this point, a voltage is applied to the structure, which induces thermal effects to control the phase state. In such a case, the structural colors could be dynamically tunable by adjusting the applied voltage. To achieve different color palettes in the visible wavelength range, we systematically varied the thickness of PMMA and VO_2_. The initial colors correspond to the structures with PMMA film of different thickness, as shown in the left column of Figure 3(b), which are significantly changed under a low voltage of 1.4 V (the middle column of Figure 3(b)). To better illustrate the color differences before and after the phase transition, the color difference Δ*E* is calculated by using the following formula:

(3) $$\Delta E=\sqrt{{\left(\Delta L^{*}\right)}^{2}+{\left(\Delta a^{*}\right)}^{2}+{\left(\Delta b^{*}\right)}^{2}},$$

where Δ*L* ^*^, Δ*a* ^*^, and Δ*b* ^*^ represent the difference between two colors on the three axes of *L* ^*^ (lightness of the color), *a* ^*^ (from green to red), and *b* ^*^ (from blue to yellow), respectively. As the results are shown in Figure 3(b), for the cavity with a thickness of approximately 90 nm, the Δ*E* value in the CIE Lab color space can reach 81. For structures with PMMA thicknesses of 70, 150, and 250 nm, the color difference exceeds 60, making the changes easily distinguishable by the human eye. Meanwhile, Figure 3(c) shows the reflection spectra for the PMMA films with thicknesses from 5 to 250 nm when VO_2_ is in the insulating and metal phases, respectively, where the experimental and simulated results are well consistent. The small deviation observed between the simulation and experimental results can be largely attributed to the inaccurate refractive index of VO_2_ thin film in simulations compared to its realistic value, as well as the experimental imperfections during measurements. It should be mentioned that by controlling the thickness of the top VO_2_ layer, one can adjust the loss within the SS cavity, thereby regulating the resultant color changes. Such a capability is well demonstrated by our experiments with VO_2_ of different thickness (40 and 80 nm in Supplementary Figures S3 and S4, respectively). As illustrated in Figure 3(d), the three data sets presented in the CIE 1931 color space demonstrate that under the same situation, the thicker the VO_2_ film, the larger the color change.

### 2.3 Performances of tunable colors

**Figure 4:**
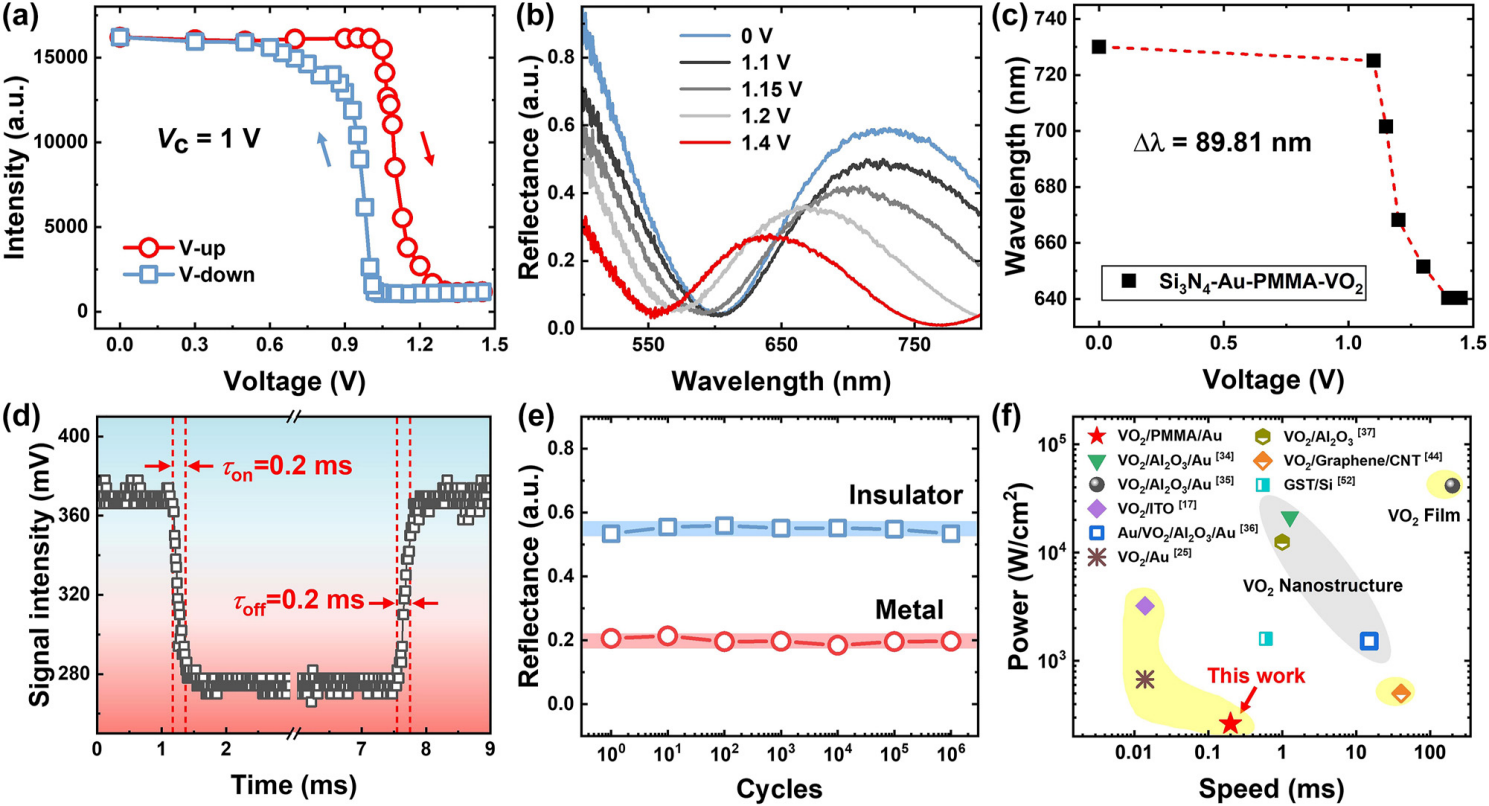
The tunable performance and the dynamic response of the SS cavities. (a) Reflection intensity of incident light at wavelength 729 nm versus external voltage. (b) Reflection spectra of VO_2_/PMMA/Au cavity with the applied voltage increasing from 0 to 1.4 V. (c) Resonance wavelength of the reflection spectra in (b) versus the applied voltage with *d*_PMMA_ = 250 nm. (d) Measured signal intensity versus time when the voltage is switched off-on-off at 1.4 V, showing a fast rising and fall time of ∼0.2 ms. (e) Measured reflectance at *λ* = 700 nm for ∼1,000,000 cycles of switching between 0 and 1 V. (f) Statistics and comparison of the response time and power consumption of electrically driven VO_2_ nanostructure, film, and GST devices in literatures and this work.

The sensitivity, speed, lifespan, and wide-view angle of electrically tunable structural colors are critical for device applications. To evaluate the sensitivity, Figure 4(a) plots the measured voltage-dependent reflection intensity of the transferred VO_2_ film at wavelength 729 nm. It can be seen that at a voltage of 0 V, the reflected light intensity is around 16,000. When the applied voltage increases to 1.4 V, the light intensity drops significantly to 1,200. At the critical voltage (*V*_c_) of 1 V, VO_2_ undergoes a fast transition from the insulator phase to the metal phase. Such a significant change results from the changes in the refractive index of VO_2_ during the phase transition, which causes changes in both spectral peaks and intensities, as shown by the voltage-dependent spectra in Figure 4(b) (also see simulation results in Supplementary Figure S1(c)). Specifically, with the increase of applied voltage, the reflection peak is gradually blue-shifted. Further evidence is provided in Figure 4(c), which illustrates the correlation between the peak wavelength and voltage. Within the voltage range of 1.1–1.4 V, the wavelength changes by 89.8 nm. Here, the sensitivity can be defined by:

(4) $$S=\frac{\Delta \lambda }{\Delta V},$$

where Δ*λ* represents the change of resonance wavelength and Δ*V* represents the change of relative voltage. In such a case, the average sensitivity of the sample is calculated to be 299.3 nm/V.

In addition, to measure the switching speed of the VO_2_-based tunable structural colors, the voltage at which each device is fully actuated is applied. As displayed in Figure 4(d), the preset structure initially displays a blue color, which then turns pink within 0.2 ms when a voltage of 1.4 V (with current 0.3 A) is applied. Once the applied voltage is removed, the color reverts to blue. Such a color change is controlled through changing the bias current, which consumes power of no more than 0.42 W. These results indicate that the ultralow thermal capacity of the freestanding VO_2_/PMMA/Au film effectively reduces the modulated voltage and response time. The rapid switching speed and low power consumption of this structure underscore its potential for advanced applications in dynamic displays and other optical technologies where fast and efficient color modulation is essential.

Furthermore, the durability of the structure is tested by applying an alternating voltage with a frequency of 50 Hz in the ambient environment. As shown in Figure 4(e), the reflection appears to be very stable under two states throughout one million switching cycles. Figure S5 shows microscope images of the pinwheel pattern on the first day (Figure S5(a)) and after 180-day storage (Figure S5(b)) in air. There is no obvious difference between the colors of the metallic phase and the insulating phase of the VO_2_/PMMA/Au structure, demonstrating excellent stable performance in air. The performance of the developed electrically controlled VO_2_ structural color device is further compared with several recently developed electrically controlled structures, as shown in Figure 4(f). It can be seen that compared with other VO_2_ nanostructures, VO_2_ thin films, and Ge_2_Sb_2_Te_5_ (GST) [52], the suspended VO_2_ nanostructure here exhibits advantages regarding both response time and power consumption.

**Figure 5:**
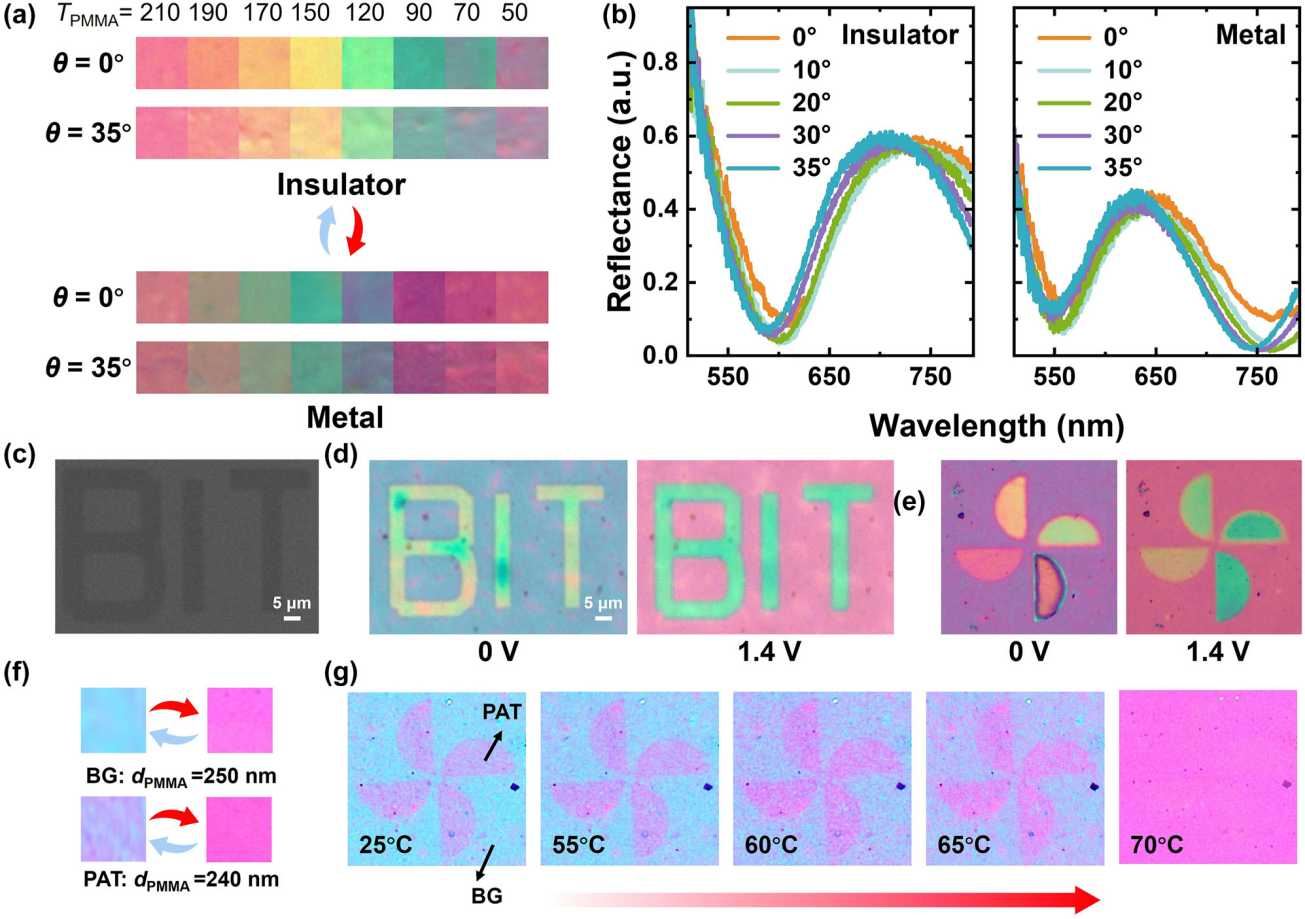
Angle-dependent performance of the nanostructure and its applications in color displays. (a) and (b) Measured images and reflection spectra of hybrid structures with PMMA of different thickness under different observation angles (*θ*) in the insulating and metal phase of VO_2_, respectively. (c) SEM image of a “BIT” pattern prepared after the transfer of VO_2_ film. (d) Optical images of “BIT” patterns at the insulating (right) and metal (left) phase of VO_2_ when turned on and off at 1.4 V. Structural parameters: *d*_PMMA_ = 150 nm, *d*_VO2_ = 120 nm. (e) Optical images of a pinwheel pattern obtained by grayscale exposure before and after the phase change actuate by external voltage of 1.4 V. (f) Optical images of two squares with PMMA thickness of 250 and 240 nm in the background (BG) area and pattern (PAT) area, respectively (*d*_VO2_ = 120 nm). The initially different colors “converge” to the same color after the phase change. (g) Temperature-dependent evolution of the color of the pinwheel pattern when temperature increases from 25 to 70 °C, where the contrast of the image decreases and finally the image is “erased.”

It should be mentioned that various visual experiences and scenarios often require consistent color displays from wide angles. To investigate the angle-dependent performance of the color displays, the VO_2_/PMMA/Au nanostructures with different thickness are characterized. As shown in Figure 5(a), the microscopic images of the nanostructures show nearly the same colors when at observation angles *θ* = 0° and *θ* = 35° at each thickness. This is also verified by the measured angular reflection spectra (0–35°) in Figure 5(b) (*d*_PMMA_ = 250 nm, *d*_VO2_ = 120 nm), demonstrating that the displayed colors are consistent with only slight changes at angles from 0° to 35°. When VO_2_ is in the insulating phase, the resonance valley of the reflection spectrum blue-shifts from 602 nm (0°) to 584 nm (35°), and the relative variation of resonant wavelength (Δ*λ*_R_/*λ*_0_) is only 2.99 %. As for the metallic phase VO_2_, the resonance valley blue-shifts from 557 nm (0°) to 548 nm (35°), and the relative variation is reduced to 1.62 %. This small shift in resonant wavelength does not cause a significant change in color perception. Therefore, unless the observation angle is large enough (for example >40°), the angle-dependent shift in resonant wavelength and the resulting change in color remain small, which is verified by the simulation results in Figure S6 (see more details in Supporting Information). Such characteristic makes the nanostructures suitable for practical applications where light sources may impinge at various angles, thereby enhancing their applicability in real-world dynamic color display technologies.

### 2.4 Applications in color displays

The ability of the grayscale exposure to independently and precisely adjust pixel heights allows for the production of structured color graphics. In this case, Figure 5(c) shows the scanning electron microscope (SEM) image of a sample with the pattern “BIT,” where the thickness of PMMA and VO_2_ are 150 and 120 nm, respectively, and the background area remains unexposed. Such a pattern shows stable color changes between 0 and 1.4 V when the phase transition of VO_2_ occurs, as shown in Figure 5(d). In addition, by varying the electron beam exposure dose, patterns with PMMA of different thickness can be designed. As shown in Figure 5(e), a colorful pinwheel is formed, with four blades showing their own structural colors. As the phase change occurs, the color of each blade in the pinwheel pattern dynamically changes, showing the versatility of structured color graphics achieved through this approach.

Another interesting phenomenon is that for some structures with different initial colors (with PMMA of 10-nm difference in thickness) at the insulator state, the same colors are displayed after the phase change at the metal state, as shown in Figure 5(f). Since VO_2_ is sensitive to temperature, such a “convergence” effect in the color display can be employed for information erasing purposes or to monitor the temperature of the environment. For example, in Figure 5(g), the contrast of the image decreases with the increase of the temperature and the image is finally invisible when the temperature is higher than 70 °C. Therefore, by carefully controlling the PMMA thickness and exposure dose, the method not only achieves dynamic color tuning for aesthetic and decorative purposes but also enables functionalities such as anticounterfeiting, information erasing, and information encryption. This precise control over structural colors underscores the versatility of our approach, making it suitable for a wide range of applications beyond conventional display technologies. It should be mentioned that with the high dielectric constant of the spacer layer, the lossless dielectric materials could produce stronger effects in optical interferences, resulting in more vivid and saturated colors. In the future, by applying etching-based fabrication methods on the spacing materials, the high-dielectric-constant materials can be integrated into these systems. Therefore, this approach not only offers greater flexibility in material selection but also enables the combination of new materials. Such advancements may enable novel optical properties and functionalities for various applications.

## 3 Conclusions

In conclusion, a simple nanofabrication method with EBL-based grayscale exposure and VO_2_-based conformal coating was demonstrated. The VO_2_ layers were coated onto the PMMA spacer layers of variable thickness, with gold films in the bottom to create SS cavities with different resonant wavelengths. Versatile VO_2_-based structural colors were constructed, exhibiting high performance of dynamically tunable properties, such as a short switching time of 0.2 ms when actuated by a low voltage of 1.4 V. The applications of color displays and information erasing were briefly demonstrated. These findings highlight the great potential of our approach for dynamic tuning of structural colors and information encryption applications, offering a wide range of possibilities for development in these fields.

## 4 Methods

### 4.1 Numerical modeling

The optical responses of the structures were simulated by using the finite element method. Optical reflection spectra were obtained under light incidence propagating along the *z*-axis direction. Periodic boundary conditions were applied on a unit cell in the *x* and *y* directions. The optical properties of Au and Si_3_N_4_ were obtained using data from Johnson and Christy and Vogt, respectively. The refractive index of PMMA was set to 1.49. Additionally, Figure S1(a) presents the wavelength-dependent refractive index (*n*) and absorption coefficient (*k*) of VO_2_ thin films in both insulating and metallic phases, as measured by ellipsometry.

### 4.2 Sample fabrications

#### 4.2.1 Synthesis of the VO_2_ film on SiO_2_ substrate

To prepare the freestanding VO_2_ films, magnetron sputtering was firstly employed to deposit high-purity vanadium to form the VO_*x*_ layer on the SiO_2_ substrate. Sputtering was performed at 0.6 Pa with a flowing gas mixture (30 sccm Ar and 20 sccm Ar/O_2_ mixture (O_2_:4 %), DC power of 55 W) at 25 °C. After VO_*x*_ deposition, the VO_*x*_ film was annealed in a low-pressure oxygen atmosphere (4.5 Pa) at 450 °C for 10 min.

#### 4.2.2 Device fabrications

To prepare the SS cavity based on VO_2_/PMMA/Au film, a layer of 100-nm-thick Au electrodes was first lithographically patterned and deposited on the Si_3_N_4_ window (Beijing ZXBR Technology Co. Ltd, 100 nm) with a window area of 400 × 400 µm by using E-beam evaporation. After the residual metal was lifted off in NMP at 60 °C, the PMMA (Allresist, 950 K) resist was then spin-coated (6,000 rpm, 30 s) with a thickness of 250 nm. Subsequently, the patterns with different heights were exposed under varied doses by EBL (20 kV, 0.3 nA, 20 μm aperture), while the irradiation dose can be selected from 0 to 120 μC/cm^2^. The samples were then immersed in MIBK solution at 10 °C for 40 s to remove the exposed resist. After that, the PMMA film was treated by oxygen plasma (100 sccm O_2_ for 10 s, power of 50 W) to enhance its hydrophilicity. As shown in Figure S2, a buffered oxide etch (BOE, 5:1 vol ratio of 40 % NH_4_F to 49 % HF) solution was used to wet etch the VO_2_/SiO_2_ interface to obtain a freestanding VO_2_ film. After etching the VO_2_/SiO_2_ interface layer, the VO_2_ layer was separated from the SiO_2_ substrate and transferred to deionized water. Finally, the released VO_2_ film was conformally transferred onto the PMMA surface, as verified by the AFM characterizations.

### 4.3 Material characterizations

A home-built optical system was utilized to measure the reflection spectra. The stabilized tungsten-halogen light source (360–2,600 nm, HL-2000, Ocean Optics) was launched into the microscopic system through a fiber with a core diameter of 100 μm. An objective lens (×10 with NA = 0.3, Olympus) was used to focus the incident light onto the sample. The reflected light in the normal direction was collected by the same objective and delivered to a spectrometer (400–1,000 nm, PG2000-Pro, Ideaoptics). Angle-resolved reflectance was characterized by the micro-angle-resolved spectroscopy system (−60°–60°, ARMS, Ideaoptics). An optical microscope (BX FM, Olympus) was used to take the optical images where the phase change was controlled by a hot plate and a source meter. Sample temperatures could be tuned precisely by a hot and cold stage (20–600 °C, HCS621GXY, INSTEC). For the electric control test, the DC voltage was supplied by a source meter (2,450, Keithley). The dynamic time response of the reflection intensity was obtained from the output of the biased Si detectors (200–1,100 nm, DET10A2, Thorlabs) and recorded by an oscilloscope (TBS2000 Series, Tektronix). AFM imaging was performed by an atomic force microscope (WITec, alpha 300).

## Supporting Information

See the supplementary material for additional information.

## Supplementary Material

Supplementary Material Details
