# Supplementary material for: Tunable structural colors based on grayscale lithography and conformal coating of VO2
Source: Nanophotonics. 2025 Jan 17;14(8):1123–33. doi: 10.1515/nanoph-2024-0546 (PMC12019934; doi:10.1515/nanoph-2024-0546)
Supplement: Supplementary file 1 — Supplementary Material Details [file j_nanoph-2024-0546_suppl_001.docx]

**Supplementary Information**

**Tunable structural colors based on grayscale lithography and conformal coating of VO_2_**

Xiaochen Zhang,^1^ Haozhe Sun,^1^ Yuan Li,^2^ Jianhua Hao,^2^ Qinghua Liang,^1^ Yongyue Zhang,^1^Yang Wang,^1^ Xiaowei Li,^3^ Xinping Zhang,^2^ He Ma,^2*^ Jiafang Li^1*^

*^1^ Key Lab of Advanced Optoelectronic Quantum Architecture and Measurement (Ministry of Education), Beijing Key Lab of Nanophotonics & Ultrafine Optoelectronic Systems, and School of Physics, Beijing Institute of Technology, Beijing 100081, China*

*^2^ Institute of Information Photonics Technology, School of Physics and Optoelectronic Engineering, Beijing University of Technology, Beijing 100124, China*

*^3^ Laser Micro/Nano Fabrication Laboratory, School of Mechanical Engineering, Beijing Institute of Technology, Beijing 100081, China*

*** [*mahe_beijing@bjut.edu.cn*](mailto:mahe_beijing@bjut.edu.cn)*;* [*jiafangli@bit.edu.cn*](mailto:jiafangli@bit.edu.cn)

**
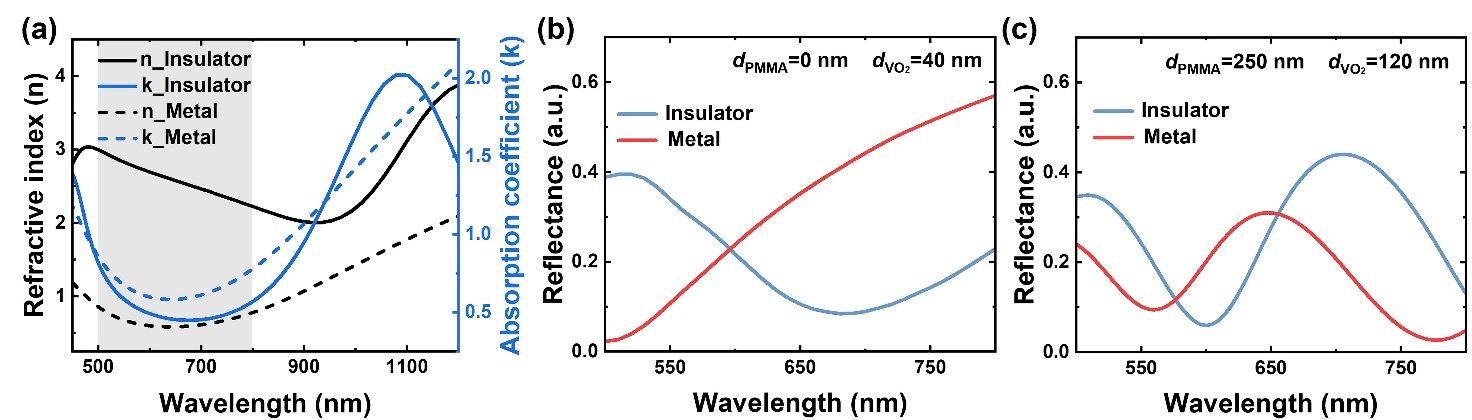
**

**Figure S1:** (a) Measured refractive index (n) and absorption coefficient (k) as a function of wavelength, which is extracted from the spectroscopic ellipsometry measurements of VO_2_ thin films deposited on SiO_2_ substrates by magnetron sputtering. (b) and (c) Simulated reflection spectra of insulating and metallic phases of a 40 nm VO_2_/0 nm PMMA/100 nm Au structure and 120 nm VO_2_/250 nm PMMA/100 nm Au structure, respectively.


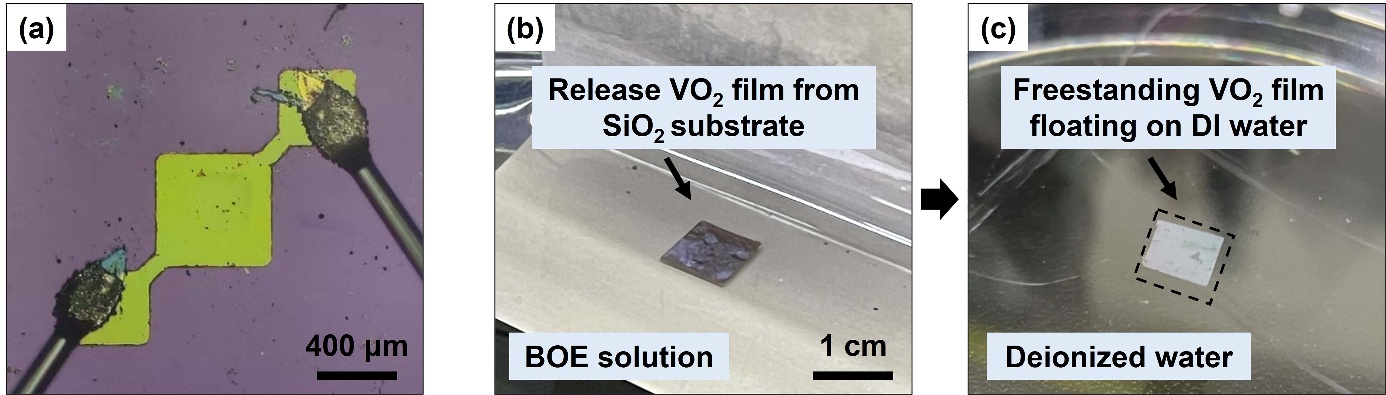


**Figure S2:** (a) Microscope image of an electrode pattern on Si_3_N_4_ window fabricated by laser direct writing. (b) VO_2_ film is prepared on the SiO_2_ structure via reactive magnetic sputtering and a post-annealing process, and the VO_2_/SiO_2_ interface can be removed by using the BOE solution. (c) Freestanding VO_2_ film floating on deionized water (DI water) after wet etching process.

**
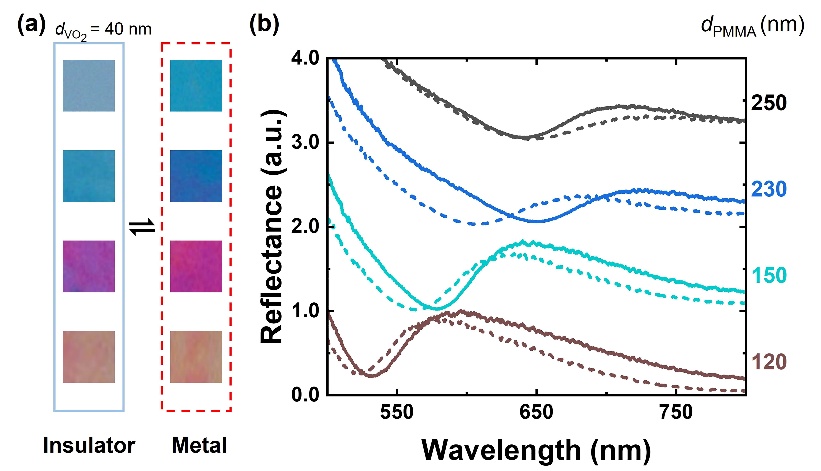
**

**Figure S3:** (a) Color images of the squares with PMMA of different thicknesses when the 40-nm-thick VO_2_ film is in the insulating and metal phases, respectively. The blue solid frame and red dotted line frame represent the insulating and metal phases of VO_2_, respectively. (b) Measured reflection spectra of an FP cavity composed of a 40 nm VO_2_ film, a PMMA film of varying thicknesses, and a 100 nm gold film.

**
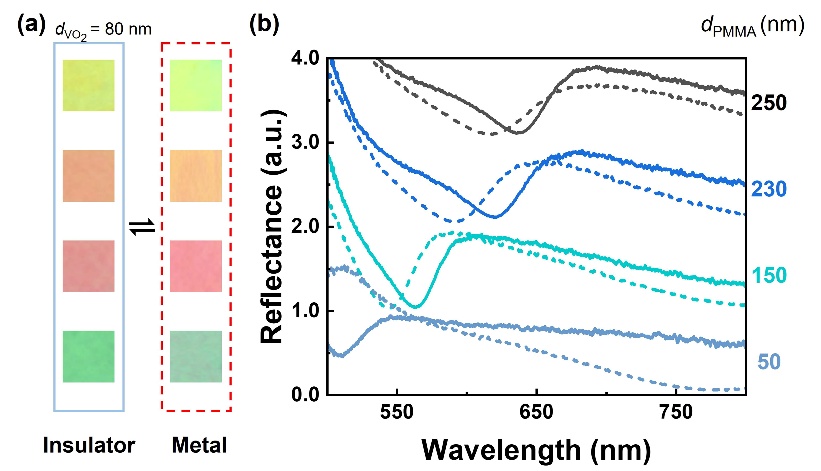
**

**Figure S****4:** (a) Color images of the squares with PMMA of different thicknesses when the 80-nm-thick VO_2_ film is in the insulating and metal phases, respectively. The blue solid frame and red dotted line frame represent the insulating and metal phases of VO_2_, respectively. (b) Measured reflection spectra of an FP cavity composed of an 80 nm VO_2_ film, a PMMA film of varying thicknesses, and a 100 nm gold film.


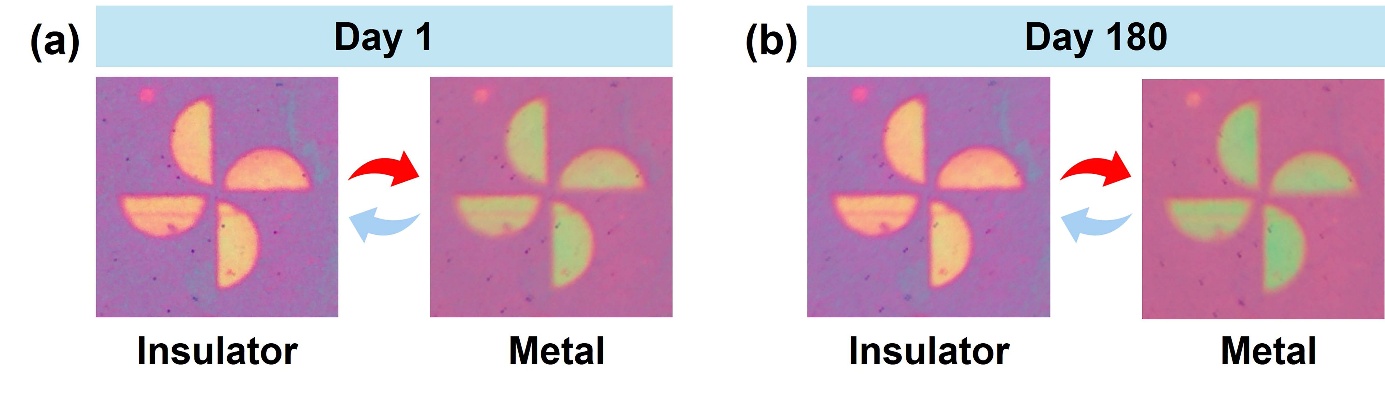


**Figure S5:** (a) and (b) Microscope images of the pinwheel pattern on the first day (a) and after 180 days (b) of storage in air.


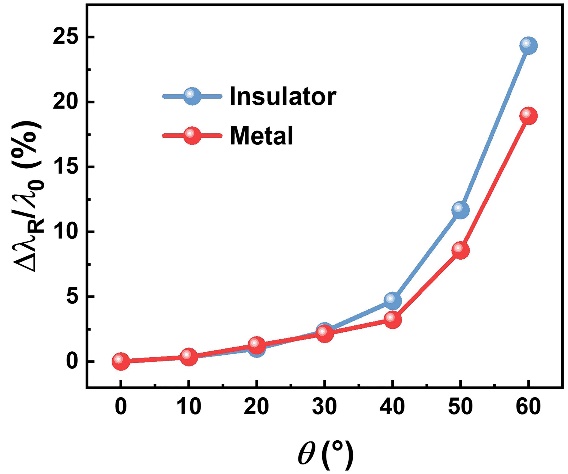


**Figure S6:** Simulated relative variation in resonance wavelength (Δ*λ*_R_/*λ*_0_) versus observation angles from 0° to 60°. Δ*λ*_R_ represents the shift in peak wavelength position, and *λ*_0_ represents the peak position at *θ*=0 for the structure with *d*_PMMA_=250 nm, *d*_VO2_=120 nm.

Figure S6 illustrates the calculated variation in resonance wavelength as a function of the observation angle in the FP structure. When the observation angle is below 40°, the variation in resonance wavelength remains less than 4.5%. In contrast, as the θ increases above 40°, the resonance wavelength shifts more significantly.
